# Atherogenic Index of Plasma Is a Potential Biomarker for Severe Acute Pancreatitis: A Prospective Observational Study

**DOI:** 10.3390/jcm9092982

**Published:** 2020-09-15

**Authors:** Seung Kook Cho, Jae Woo Kim, Ji Hye Huh, Kyong Joo Lee

**Affiliations:** 1 Department of Internal Medicine, Gangwondo Wonju Medical Center, Wonju 26448, Korea; lukechosk@gmail.com; 2 Department of Internal Medicine, Yonsei University Wonju College of Medicine, Wonju 26426, Korea; jawkim96@yonsei.ac.kr; 3 Division of Endocrinology and Metabolism, Department of Internal Medicine, Hallym University Sacred Heart Hospital, Anyang 14068, Korea

**Keywords:** acute pancreatitis, atherogenic index of plasma, biomarker, severity, Atlanta classification

## Abstract

Background: The atherogenic index of plasma (AIP) reflects the levels of triglycerides (TG) and high-density lipoprotein (HDL) cholesterol. The purpose of this study was to assess the relationship between the AIP and severe acute pancreatitis (SAP). Materials and methods: Patients with acute pancreatitis (AP) were prospectively enrolled from March 2015 to June 2019. The severity of AP was classified according to the 2012 revised Atlanta classification. Mild and moderately severe AP were categorized as non-SAP. The AIP is calculated as log(TG/HDL). Results: A total of 323 patients were enrolled. The etiologies of AP were gallstone in 171 patients (52.9%), alcohol in 122 patients (37.8%), and hypertriglyceridemia in 30 patients (9.3%). Twenty-four patients (7.4%) were classified as SAP. The AIP was significantly higher in the SAP group compared to the non-SAP group (*p* < 0.001). The AIP was positively correlated with the Atlanta classification (*R* = 0.256, *p* < 0.001). In multivariate analysis, the AIP was found to be an independent predictive factor for SAP (OR = 4.571; CI = 1.913–10.922; *p* = 0.001). Conclusions: The AIP is a potential biomarker for the prediction of SAP in clinical practice. This result provides that impaired lipid metabolism is associated with the severity of pancreatitis.

## 1. Introduction

Acute pancreatitis (AP) is an inflammatory disease of the pancreas which can cause local injury, systemic inflammatory response syndrome (SIRS), and organ failure (OF). The incidence of AP is increasing, with an annual incidence of 5–30 per 100,000 individuals [1,2]. The overall mortality rate for AP is approximately 5%, and is up to 30% in patients who experience SIRS and OF [3]. According to the 2012 revised Atlanta classification, severe acute pancreatitis (SAP) is defined when there is persistent OF (>48 h) [4]. The predicted mortality of SAP is approximately 10%, as compared with less than 1% in patients whose AP is predicted to be mild [5]. Therefore, the prediction of the severity of AP is crucial to triage patients during the initial diagnosis. Early intensive care is necessary for patients with SAP in order to reduce the morbidity and mortality. Complex scoring systems have been developed to determine the severity of AP, including the Ranson scoring system, Acute Physiology and Chronic Health Evaluation (APACHE) II, computed tomography scoring index (CTSI), and Bedside Index for the Severity in Acute Pancreatitis (BISAP) [6,7,8,9]. Inflammatory markers such as C-reactive protein (CRP) or procalcitonin are also used as single parameters for the prediction of the severity of AP [10].

The atherogenic index of plasma (AIP) reflects the levels of triglycerides (TG) and high-density lipoprotein (HDL) cholesterol, and is calculated as log(TG/HDL). The AIP has been used to quantify blood lipid levels and as a robust biomarker of dyslipidemia and atherogenicity. It is also considered an indicator of metabolic syndrome and coronary syndrome [11,12]. In this regard, local and systemic lipotoxicity has been recognized as an important risk factor for SAP or multisystem OF in AP [13]. Previous studies have reported that low HDL cholesterol is an independent predictor for SAP and longer hospitalization for patients with AP [14,15]. Elevated serum TG is also a well-known risk factor of persistent OF in AP [16]. Considering the close relationship between impaired lipid metabolism and SAP, we assume that the AIP may be a predictor of the severity of AP.

However, the relationship between the AIP and the severity of AP remains unknown. Therefore, we determined the relationship between the AIP and the severity of AP and evaluated the predictive ability of the AIP for the severity of AP compared with other scoring systems.

## 2. Materials and Methods

### 2.1. Study Population

Patients diagnosed with AP at Yonsei University Wonju College of Medicine between March 2015 and June 2019 were enrolled in this prospective observational study. The study protocol was approved by the Internal Review Board for Human Research of Yonsei University Wonju College of Medicine (approval no. CR315005-002). This study was performed in accordance with the relevant guidelines and regulations. Written informed consent was obtained from all patients.

### 2.2. Diagnostic Criteria

The diagnosis of AP was based on the presence of two of the following three criteria: (1) upper abdominal pain, (2) serum amylase and/or lipase ≥3 times the upper normal limit, and (3) characteristic imaging on a radiological study [17]. The etiology of AP included gallstones, alcohol consumption, and high TG. The exclusion criteria were the following: (1) age < 18 years old, (2) idiopathic pancreatitis, (3) patients with incomplete lipid profile data, (4) chronic pancreatitis, and (5) recurrent pancreatitis.

### 2.3. Data Collection

Clinical data and blood samples were obtained upon the patients’ diagnoses of AP. The presence of hypertension, diabetes mellitus, alcohol consumption, and smoking was recorded. The patients’ body mass index (BMI) was calculated using their height and weight. Hemoglobin, hematocrit, white blood cell (WBC) count, blood urea nitrogen, creatinine, lactate dehydrogenase, aspartate aminotransferase, CRP, procalcitonin, amylase, and lipase were recorded at the time of admission. Abdominal computed tomography (CT) scans were performed to diagnose AP and differentiate AP from other diseases. Once AP was diagnosed, the TG and HDL levels were measured as soon as possible. Additionally, scoring systems such as the Ranson score, BISAP, and CTSI were calculated. The AIP was defined as log (TG/HDL). The severity of AP was evaluated according to the 2012 revised Atlanta classification and recorded as mild, moderately severe, or severe AP [4]. Mild AP is defined by the absence of OF and local or systemic complications. Moderately severe AP is described as transient OF that resolves within 48 h. SAP is described as persistent OF. Non-SAP included mild AP and moderately severe AP.

### 2.4. Statistical Analysis

Categorical variables were presented as frequencies and percentages. Continuous variables were presented as means (± standard deviation) or medians with ranges. The Pearson rank method was used to evaluate the correlation between the AIP and the Atlanta classification. After adjustments for confounding factors, the odds ratios for SAP were evaluated using univariate and multivariate logistic regression analyses in order to determine the predictive factors of SAP among all the patients with AP and the predictive factors for intensive care unit (ICU) admission. The accuracy of the prediction power for SAP was determined by the area under the curve (AUC) using receiver operating characteristic (ROC) curves. AUC values were used to compare the predictive abilities of SAP using the AIP and scoring systems. A *p* value < 0.05 was considered statistically significant. Analyses were performed using SPSS version 23 (IBM, Armonk, NY, USA).

## 3. Results

### 3.1. Baseline Characteristics of Patients

**Figure 1:**
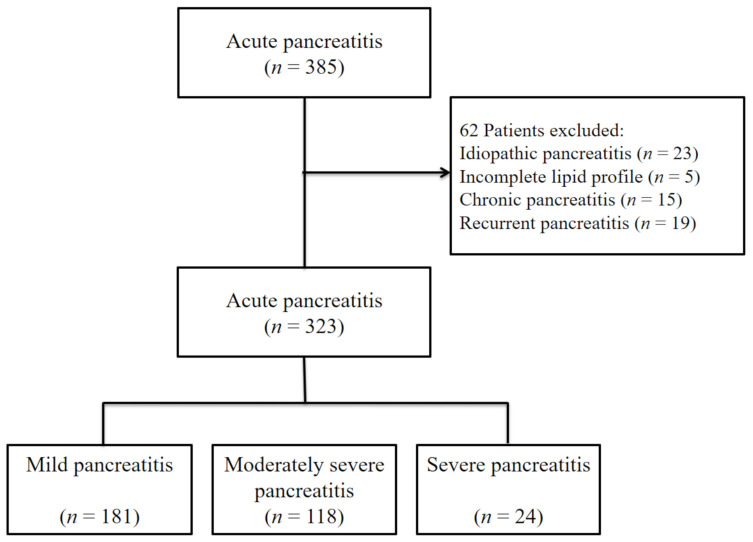
Flow chart of the study population.

A total of 323 patients were included in this study (Figure 1). Table 1 describes the characteristics of the participants. The mean age was 56.2 ± 18.3 years, and 271 (68.8%) patients were male. The etiologies of AP were gallstones in 171 patients (52.9%), alcohol over-consumption in 122 patients (37.8%), and hypertriglyceridemia in 30 patients (9.3%). According to the 2012 revised Atlanta classification, the severity of AP was mild in 181 patients (56%), moderately severe in 118 patients (36.5%), and severe in 24 patients (7.4%). The Ranson score, CTSI, and BISAP were higher in the SAP group compared to the non-SAP group (*p* < 0.001). The number of ICU admissions, mortality, and the duration of hospital stays were significantly higher in the SAP group compared to the non-SAP group (*p* < 0.001). The levels of CRP, procalcitonin, HDL, and TG were not different between the two groups, but the level of the AIP was significantly higher in the SAP group (1.0 ± 0.6 vs. 0.5 ± 0.5, *p* < 0.001).

### 3.2. The AIP and Scoring Systems for Predicting SAP

**Figure 2:**
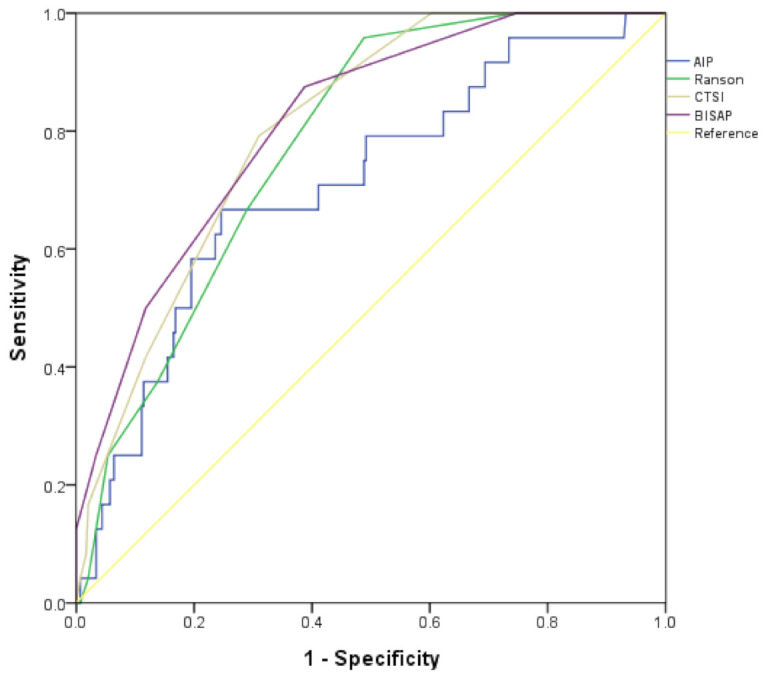
Receiver operator characteristic curve of various factors as predictors of severe acute pancreatitis. AIP: atherogenic index of plasma; CTSI: computed tomography scoring index; BISAP: the Bedside Index for Severity in Acute Pancreatitis.

We calculated the AUCs of various scoring systems and the AIP for predicting SAP (Table 2). The AUC of BISAP showed the greatest accuracy for the prediction of SAP (AUC = 0.813). The AUC of the AIP for prediction of SAP was 0.709, which was not significantly different from the AUC of other scoring systems (Figure 2). The AIP was positively correlated with WBC (*R* = 0.146; *p* = 0.009), CRP (*R* = 0.236; *p* < 0.001), and BMI (*R* = 0.134; *p* = 0.016) (Table 3). Furthermore, the AIP was positively correlated with the Atlanta classification (*R* = 0.256, *p* < 0.001).

### 3.3. Predictive Factors Affecting SAP and ICU Admission

In the univariate analysis, the procalcitonin (OR = 1.015; *p* = 0.008) and AIP (OR = 3.119; *p* < 0.001) were related to SAP. In the multivariate analysis, the AIP (OR = 4.571; CI = 1.913–10.922; *p* = 0.001), alcohol (OR = 5.782; CI = 1.111−30.088; *p* = 0.037), and procalcitonin (OR = 1.013; CI = 1.000–1.025; *p* = 0.043) were independent predictive factors for SAP (Table 4). Univariate analysis revealed that ICU admission is related to alcohol, CRP, procalcitonin, and the AIP. In multivariate analysis, the AIP (OR = 5.099; CI = 2.340–11.112; *p* < 0.001), alcohol, and procalcitonin were independent predictive factors for ICU admission.

## 4. Discussion

In this cross-sectional study, we explored the relationship between the AIP and the severity of AP, and we found that the AIP is higher in patients with severe AP compared to patients with mild or moderately severe AP. We also found that the AIP is positively correlated with the 2012 revised Atlanta classification. Furthermore, the AIP is an independent predictor of SAP, and the predictive ability for SAP is superior to that of either of TG or HDL cholesterol. These results suggest that the AIP may be a simple biomarker for predicting SAP. To our knowledge, this is the first study to evaluate the relationship between the AIP and AP.

Many studies have focused on the early diagnosis and treatment of SAP in order to reduce the high morbidity and mortality of AP. A diagnosis of SAP is based on clinical manifestations, laboratory tests, and imaging. However, it is difficult to predict the course of the disease, as AP is complex and the clinical course varies even when the clinical and radiological scores are the same at the initial diagnosis [18]. Traditional scoring systems are complicated and time consuming, as they are calculated based on several clinical findings and laboratory parameters. Many investigators have tried to find simple and convenient biomarkers to predict the severity of AP. Inflammatory markers, including CRP and procalcitonin, and various cytokines and chemokines, including tumor necrosis factor-α and interleukin-6, have been evaluated as predictors of SAP, as well as markers of the development of specific OF [19]. Recently, several studies investigated the association between AP and dyslipidemia. Morbid obesity and the presence of metabolic syndrome have shown a higher risk of moderately severe AP and SAP [20,21]. These results suggest that an indicator for both dyslipidemia and inflammatory status could be the ideal biomarker for predicting the severity of AP. The AIP is a comprehensive lipid index and is used as a reasonable marker for both inflammation and impaired lipid metabolism [22,23].

Our study illustrates several important and novel findings. First, there were correlations between an increased AIP and significant increases in the WBC count and serum CRP. As pancreatic inflammation is the most important characteristic feature of AP and involves an excessive recruitment of leukocytes [24], the levels of WBC and CRP have a major role in AP. The WBC count is included in the Ranson score and is one of the clinical criteria of systemic inflammatory response syndrome [6]. Additionally, the relationship between coagulation and inflammation are two-way and are based on positive feedback. Therefore, the development of inflammation leads to the activation of coagulation [25]. The markers of activated coagulation are useful in the early prediction of SAP [26]. Additionally, a previous study has evaluated the predictive value of CRP in AP [27]. Second, the AIP was positively correlated with BMI in our study. Obesity has roles in initiating AP and worsening AP outcomes [28]. Obesity is related with increased gallbladder stones, hypertriglyceridemia, and diabetes. Furthermore, increased intrapancreatic and peripancreatic fat induce local pancreatic necrosis and systemic injury, including OF [29]. Third, the AIP was positively correlated with the 2012 revised Atlanta classification in this study. As this classification system is widely used for the severity stratification of AP, this correlation suggests that a higher AIP might be related to SAP. Finally, the AIP was an independent predictive factor for SAP and ICU admission in this study. This result is in accordance with our hypothesis. As the AIP is easily calculated with the level of TG and HDL, it is a useful biomarker to predict the severity of AP in clinical practice.

The AIP reflects the ratio of serum TG and HDL cholesterol and is calculated as log(TG/HDL). Hypertriglyceridemia is a common etiology of AP, and elevated serum TG is independently associated with persistent OF [16,30]. Hypertriglyceridemia-induced pancreatitis is not fully understood, but hypertriglyceridemia is thought to increase plasma viscosity, induce pancreatic ischemia, and trigger organ inflammation [31]. In contrast, decreased HDL cholesterol is related with pancreatic necrosis, persistent OF, and mortality in AP [14,15]. Low HDL, which has anti-inflammatory properties, can lead to a more severe systemic inflammatory response [32]. The AIP has been suggested to be a surrogate of small, dense low-density lipoprotein cholesterol, which plays a central role in the induction of pro-inflammatory mediators and the reduction in inducing anti-oxidants [33]. Therefore, the predictive power of the AIP for SAP may be due to the fact that the AIP reflects the interaction between atherogenic and protective lipoprotein. Considering the mechanism of AP pathogenesis, a higher AIP can be a useful biomarker for the prediction of the severity of AP.

There are several limitations to our study. First, the number of SAP cases was small, and the study was conducted at a single institution. However, we enrolled sufficient numbers of all AP severities with different etiologies. Second, as the design of the study was cross-sectional, we cannot determine a cause-and-effect relationship between the AIP and the severity of AP. Finally, inflammatory cytokines such as tumor necrosis factor-α and interleukin-6 were not measured. However, this study is the first study to demonstrate that the AIP is related to SAP. A large-scale study is needed to establish the clinical role of the AIP for predicting the severity of AP and providing prognoses.

In conclusion, we found that the AIP is significantly associated with SAP and that the AIP is a superior predictive factor to TG or HDL cholesterol level alone for predicting the risk of SAP. Our findings provide robust evidence regarding the association of impaired lipid metabolism and the severity of AP, thereby suggesting that the AIP is a simple and novel biomarker for predicting SAP. As the AIP can be easily measured in a convenient manner, it can be applied in clinical practice. 

## Figures and Tables

**Table 1:** Baseline characteristics of all the patients.

Variable	N = 323	SAP (*n* = 24)	Non-SAP (*n* = 299)	*p*-Value
Gender (Male: Female)	271(68.8%):123(31.2%)	14(58.3%):10(41.7%)	209(69.9%):90(30.1%)	0.238
Age, years	56.2 ± 18.3	54.7 ± 21.4	56.3 ± 18.1	0.689
Etiology of acute pancreatitis				0.134
Gallstone	171 (52.9%)	8 (33.3%)	163 (54.5%)	
Alcohol	122 (37.8%)	13 (54.2%)	109 (36.5%)	
Hypertriglyceridemia	30 (9.3%)	3 (12.5%)	27 (9%)	
Smoking	143 (44.3%)	12 (50%)	131 (43.8%)	0.557
Hypertension	124 (38.4%)	10 (41.7%)	114 (38.1%)	0.732
Diabetes Mellitus	87 (26.9%)	6 (25%)	81 (27.1%)	0.824
Body mass index, kg/m^2^	24.3 ± 4.6	25.6 ± 5.4	24.2 ± 4.5	0.162
Atlanta classification				
Mild	181 (56%)			
Moderately severe	118 (36.5%)			
Severe	24 (7.4%)			
Ranson	2.7 ± 1.6	4.2 ± 1.3	2.6 ± 1.6	<0.001
CTSI	2.1 ± 1.4	3.5 ± 1.4	1.9 ± 1.3	<0.001
BISAP	1.3 ± 1.1	2.7 ± 1.2	1.2 ± 1.0	<0.001
Hospital stay, days	6.5 ± 5.5	13.2 ± 7.1	6.0 ± 5.0	<0.001
Intensive care unit admission	40 (12.4%)	14 (58.3%)	26 (8.7%)	<0.001
Mortality	6 (1.9%)	5 (20.8%)	1 (0.3%)	<0.001
Laboratory findings				
C-reactive protein, mg/dL	4.6 ± 7.1	6.4 ± 9.6	4.5 ± 6.8	0.355
Procalcitonin, ng/mL	5.4 ± 21.8	19.8 ± 52.6	4.3 ± 16.8	0.163
HDL, mg/dL	37.7 ± 18.3	33.9 ± 18.9	38.0 ± 18.3	0.296
Triglycerides, mg/dL	302.1 ± 723	544.3 ± 860.5	282.7 ± 709.2	0.088
AIP	0.6 ± 0.5	1.0 ± 0.6	0.5 ± 0.5	<0.001

Results are presented as the mean ± standard deviation or median; CTSI: computed tomography severity index; BISAP: the Bedside Index for Severity in Acute Pancreatitis; HDL: high-density lipoprotein; AIP: atherogenic index of plasma.

**Table 2:** Area under the curve for predicting severe acute pancreatitis.

Variable	AUC	Standard Error	95% CI	*p*-Value
AIP	0.709	0.056	0.600–0.819	0.001
TG	0.683	0.040	0.606–0.761	0.003
HDL	0.371	0.057	0.260–0.482	0.035
CTSI	0.805	0.037	0.734–0.877	<0.001
Ranson score	0.778	0.038	0.704–0.852	<0.001
BISAP	0.813	0.040	0.734–0.892	<0.001

AUC: area under the curve; CI: confidence of interval; AIP: atherogenic index of plasma; TG: triglycerides; HDL: high-density lipoprotein; CTSI: computed tomography severity index; BISAP: the Bedside Index for Severity in Acute Pancreatitis.

**Table 3:** The correlation of the AIP and other variables.

Statistics	Body Mass Index	White Blood Cell	C-Reactive Protein	Atlanta Classification
AIP				
*R*	0.134	0.146	0.236	0.256
*p*-Value	0.016	0.009	<0.001	<0.001

Pearson rank method was used. AIP: atherogenic index of plasma.

**Table 4:** The association between the AIP and severe acute pancreatitis.

Variable	OR	*p*-Value *	OR	95% CI	*p*-Value ^#^
Gender (Male)	0.603	0.242	0.406	0.152–1.084	0.406
Age	0.995	0.688	1.006	0.975–1.038	0.708
Gallstone	0.417	0.051	2.069	0.288–14.870	0.470
Alcohol	2.060	0.090	5.782	1.111–30.088	0.037
Smoking	1.282	0.558			
Hypertension	1.159	0.732			
Diabetes mellitus	0.897	0.824			
Body mass index	1.067	0.157	1.077	0.975–1.189	0.143
C-reactive protein	1.031	0.214			
Procalcitonin	1.015	0.008	1.013	1.000–1.025	0.043
AIP	3.119	<0.001	4.571	1.913–10.922	0.001

* Univariate analysis was performed; ^#^ multivariate analysis was performed; OR: odds ratio; CI: confidence interval; AIP: atherogenic index of plasma.
